# The emerging functions and clinical implications of circRNAs in acute myeloid leukaemia

**DOI:** 10.1186/s12935-025-03772-4

**Published:** 2025-04-29

**Authors:** Shuiqing Liu, Xingyu Wan, Yang Gou, Wuchen Yang, Wei Xu, Yuxuan Du, Xiangui Peng, Xiaoqi Wang, Xi Zhang

**Affiliations:** 1 Medical Center of Hematology, Xinqiao Hospital of Army Medical University, Chongqing, 400037 China; 2 Chongqing Key Laboratory of Hematology and Microenvironment, Chongqing, 400037 China; 3 State Key Laboratory of Trauma and Chemical Poisoning, Chongqing, 400037 China; 4 Jinfeng Laboratory, Chongqing, 401329 China

**Keywords:** Circular RNAs, Acute myeloid leukaemia, Functions, Clinicopathologic features, Therapy

## Abstract

Acute myeloid leukaemia (AML) is a prevalent haematologic malignancy characterized by significant heterogeneity. Despite the application of aggressive therapeutic approaches, AML remains associated with poor prognosis. Circular RNAs (circRNAs) constitute a unique class of single-stranded RNAs featuring covalently closed loop structures that are ubiquitous across species. These molecules perform crucial regulatory functions in the pathogenesis of various diseases through diverse mechanisms, including acting as miRNA sponges, interacting with DNA or proteins, and encoding functional proteins/polypeptides. Recently, numerous circRNAs have been confirmed to have aberrant expression patterns in AML patients. In particular, certain circRNAs are closely associated with specific clinicopathological characteristics and thus have great potential as diagnostic/prognostic biomarkers and therapeutic targets in AML. Herein, we systematically summarize the biogenesis, degradation, and functional mechanisms of circRNAs while highlighting their clinical relevance. We also outline a series of online databases and analytical tools available to facilitate circRNA research. Finally, we discuss the current challenges and future research priorities in this evolving field.

## Introduction

Acute myeloid leukaemia (AML) is a highly lethal haematologic malignancy characterized by the uncontrolled proliferation and differentiation arrest of immature myeloid cells [1]. Much progress has been made in AML management, with significant advances in molecular biology and therapeutic technologies [2] including optimized chemotherapy regimens [3], refined haematopoietic stem cell transplantation strategies [4–6], and novel targeted therapies such as Bcl-2 inhibitors [7], FLT3 tyrosine kinase inhibitors [7], CD33-targeting agents [8], IDH inhibitors [7], and CAR-T-cell immunotherapy [9, 10]. However, the clinical outcomes of AML remain suboptimal. Current data indicate a 5-year survival rate of barely 29.5%, and this low rate is attributed primarily to treatment failure, disease relapse, chemotherapy resistance, and limitations of the haematopoietic niche [11, 12]. These persistent challenges underscore the critical need to elucidate the mechanism underlying leukaemia initiation, maintenance and recurrence and to identify novel biomarkers for improved diagnosis, risk stratification and precision therapies [13].

Circular RNAs (circRNAs) represent a unique class of RNAs characterized by covalently closed circular structures devoid of 5’ caps and 3’ polyadenylated tails [14]. These RNAs originate predominantly through the back-splicing of linear precursor mRNAs (pre-mRNAs) [15]. They exhibit distinctive biological features, including evolutionary conservation, exceptional stability, tissue-specific expression patterns, and high abundance in mammalian cells and exosomes [14, 16, 17]. Although circRNAs were initially discovered in RNA viruses by Sanger and colleagues in 1976 [18], their significance in diseases remained poorly understood until recent breakthroughs in experimental techniques and bioinformatics analyses [19]. Recent studies have revealed that circRNAs are dysregulated in almost all cancer types [20] and can functionally modulate tumour cell proliferation, apoptosis, differentiation, invasion, metastasis, angiogenesis, autophagy, metabolism, stemness maintenance, immune escape, and drug resistance [20, 21]. Therefore, circRNAs are considered promising biomarkers and therapeutic targets for tumour treatment.

Notably, an increasing number of circRNAs have been identified as critical regulators of both the initiation and progression of AML [22, 23]. However, the precise roles and regulatory mechanisms of circRNAs in AML remain incompletely understood. Herein, we comprehensively delineate the molecular life cycle of circRNAs, including their biogenesis, degradation, regulation and function, with a particular focus on AML-associated circRNA dysregulation. We examine the expression profiles of numerous circRNAs in AML and describe their biological roles in cell proliferation, apoptosis, the cell cycle, differentiation, migration, invasion, extramedullary infiltration, ferroptosis, autophagy, stemness, drug resistance, exosomes, and the tumour microenvironment as elucidated in previous research. We also elaborate on the critical potential of these circRNAs as clinical biomarkers and innovative therapeutic targets. Additionally, we outline a series of essential online databases and tools for circRNA investigation. Finally, we discuss the current challenges and potential directions for future circRNA research. We hope that this review will help researchers achieve a better understanding of circRNAs and provide directions for further studies on circRNAs.

## Biogenesis and degradation of circRNAs

**Fig. 1:**
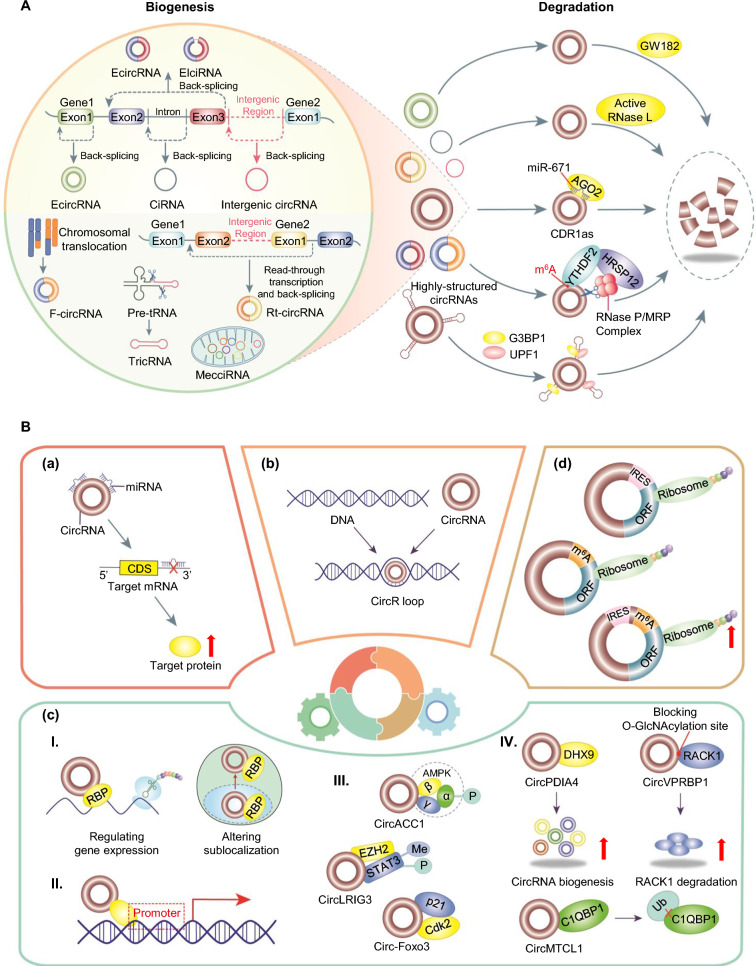
Biogenesis, degradation and regulatory functions of circRNAs. **A** Biogenesis of exonic circRNAs (ecircRNAs), exon‒intron circRNAs (EIciRNAs), intronic circRNAs (ciRNAs), intergenic circRNAs, read‒through circRNAs (rt-circRNAs), fusion circRNAs (f-circRNAs), tRNA intronic circular RNAs (tricRNAs), and mitochondria‒encoded circRNAs (mecciRNAs) and the potential mechanisms of circRNA degradation. **B** Potential regulatory mechanisms of circRNAs. (**a**) CircRNAs can serve as microRNA sponges to block microRNA-mediated target gene silencing. (**b**) CircRNAs can interact with DNA to form circR loops. (**c**) CircRNAs can regulate the functions of RBPs (I), recruit transcription activators (II), act as protein scaffolds (III), and interact with specific proteins (IV). (**d**) CircRNAs can be translated into proteins or peptides

Unlike the canonical linear splicing mechanism, circRNAs are typically produced through noncanonical alternative splicing, termed back-splicing, a process that joins downstream 5’ splice sites to upstream 3’ splice sites, forming covalently closed circRNAs [14]. Although their back-splicing efficiency is much lower than that of linear splicing, circRNAs exhibit greater stability than linear RNAs because of their circular conformation, which confers resistance to exonuclease-mediated degradation [14]. According to the literature, circRNAs that originate from pre-mRNAs can be classified into four broad categories: exonic circRNAs (ecircRNAs), exon‒intron circRNAs (EIciRNAs), intronic circRNAs (ciRNAs) and intergenic circRNAs [24] (Fig. 1A). Among them, ecircRNAs, composed exclusively of one or more exons, are the most common circRNA type and mainly localize in the cytoplasm [25]. In contrast, EIciRNAs retain intronic sequences between circularized exons and predominantly reside in the nucleus [26]. Notably, ecircRNAs and EIciRNAs may share common biogenesis mechanisms, including intron-pairing-driven circularization, RNA binding protein (RBP)-dependent circularization, or lariat-driven circularization [16, 24]. ciRNAs originate from intron lariats that fail to debranch during splicing and accumulate primarily in the nucleus [27]. Intergenic circRNAs originate from genomic regions between protein-coding genes, containing two intronic circRNA fragments that are spliced at flanking GT-AG splicing signals [28]. Moreover, several special types of circRNAs, such as fusion circRNAs (f-circRNAs) [29], tRNA intronic circular RNAs (tricRNAs) [30], mitochondria-encoded circRNAs (mecciRNAs) [31, 32], and read-through circRNAs (rt-circRNAs), have been identified [33] (Fig. 1A).

Relative to linear RNAs, circRNAs exhibit superior stability across different cell and tissue types and in exosomes [17]. However, the mechanisms of circRNA degradation remain poorly understood, although recent studies have proposed several potential degradation pathways (Fig. 1A). Collectively, the available evidence suggests that circRNAs may be degraded by GW182 (a crucial component of the P-body and RNAi machine) [34], RNase L in cells upon poly(I:C) treatment or viral infection [35], or by interacting with miRNAs in an Argonaute 2 (Ago2)-dependent manner [36]. Specifically, m^6^A-modified circRNAs undergo endoribonucleolytic cleavage through the YTHDF2 (m^6^A reader protein)-HRSP12 (adaptor protein)-RNase P/MRP (endoribonuclease) pathway [37]. Furthermore, highly structured circRNAs can be degraded by G3BP1 and its associated protein UPF1 [38]. These findings indicate that circRNA degradation involves multiple coordinated mechanisms; however, comprehensive understanding requires further investigation.

## Potential regulatory mechanisms of circRNAs

The regulatory mechanisms of circRNAs have been increasingly elucidated with research progress. Substantial evidence now confirms that circRNAs serve as critical regulators in diverse physiological and pathological processes. They exhibit multiple activities, including (1) sponging miRNAs, (2) interacting with DNA or proteins, and (3) encoding proteins or polypeptides (Fig. 1B). In this section, we systematically review the established circRNA regulatory paradigms, discuss controversial issues, and illustrate these topics with a few representative examples.

### CircRNAs as microRNA sponges

Much of the research on circRNAs to date has focused predominantly on the activities of these molecules as miRNA sponges to regulate the expression of miRNA targets [39]. For example, circRPN2 acts as a sponge of miR-183-5p to derepress the expression of FOXO1, thereby regulating glucose metabolism and metastasis in hepatocellular carcinoma [40]. CircBCAR3 promotes oesophageal cancer tumorigenesis and metastasis by sponging miR-27a-3p and thereby upregulating TNPO1 [41]. CircMETTL3 restrains colorectal cancer development and metastasis by interacting with miR-107 to increase PER3 expression [42]. However, the biological functions of circRNA-mediated miRNA sponging requires careful validation. For example, when a circRNA harbours multiple miRNA response elements (MREs) but is expressed at low levels, its capacity to function as a miRNA sponge might be limited. Similarly, circRNAs possessing few MREs and with low cellular abundance are unlikely to exert significant sponge activity. Therefore, three critical parameters require rigorous evaluation: (1) the number of MREs per circRNA molecule, (2) the stoichiometric ratio of circRNAs to miRNAs, and (3) the expression levels of the miRNA-targeted genes. Notably, sponging activity fundamentally depends on the cytoplasmic colocalization of circRNAs and their target miRNAs.

### CircRNA interact with DNA

R-loops are three-stranded nucleic acid structures comprising an RNA:DNA hybrid and single-stranded DNA that contribute significantly to DNA damage induction, genomic instability modulation, and transcriptional control [43]. Interestingly, circRNAs can form circRNA:DNA hybrids (circR loops) that critically influence malignant tumour phenotypes. A recent study demonstrated that circRNAs are enriched within leukaemia-rearranged (MLL-r) AML and can bind with DNA to form circR loops at their cognate loci [44]. In particular, circMLL(9,10) has demonstrated oncogenic potential by inducing proteasome inhibition, triggering DNA breakage, and promoting chromosomal translocation, thereby driving AML pathogenesis in vitro and in vivo [44]. Similarly, Xu et al. reported that circSMARCA5, which is expressed at reduced levels in breast cancer, forms circR loops at its parental gene locus [45]. The functional restoration of circSMARCA5 blocks SMARCA5 transcription, impairs DNA damage repair capacity, and enhances cisplatin sensitivity, suggesting that circSMARCA5 may serve as a therapeutic target in breast cancer, especially in patients with drug-resistant disease [45]. Overall, the function of circR loops in cancer is undeniably important and is usually mediated via the induction of DNA damage and genomic instability or transcription regulation. Despite these advances, the field of circR loop biology remains in its nascent exploration phase, with numerous relevant circRNAs awaiting characterization.

### CircRNA interact with proteins

#### Regulating the functions of RBPs

Emerging evidence has revealed that circRNAs interact with RBPs to regulate gene expression. For example, circMYBL2 enhances the efficiency of FLT3 kinase translation by facilitating polypyrimidine tract-binding protein 1 (PTBP1) binding to FLT3 mRNA, thereby promoting proliferation and inhibiting differentiation in FLT3-ITD-positive AML cells [46]. In cervical cancer, circTICRR is upregulated and exerts oncogenic effects by inhibiting autophagy activation through binding to HuR and stabilizing GLUD1 mRNA [47]. Moreover, circBACH1 can directly interact with HuR and alter its translocation from the nucleus to the cytoplasm, thereby inhibiting p27 mRNA translation via recognition of its interferon-responsive sequence in the 5’-untranslated region, ultimately promoting proliferation in hepatocellular carcinoma cells [48]. Overall, these findings demonstrate that circRNAs modulate RBP subcellular localization and functional activity through specific molecular interactions.

#### Recruiting transcription activators

In this section, we will present three examples that illustrate the roles of circRNAs in recruiting transcriptional activators. In hepatocellular carcinoma, the highly abundant circRNA cia-MAF recruits the histone acetyltransferase complex (TIP60) complex to the MAFF promoter, initiating transcriptional activation that sustains liver tumour-initiating cell (TIC) self-renewal [49]. Similarly, circACTN4 facilitates Y-box binding protein 1 (YBX1) recruitment to activate FZD7 transcription and then activates the Wnt and Hippo signalling pathways, thereby promoting intrahepatic cholangiocarcinoma (ICC) growth and metastasis [50]. Furthermore, circ-DONSON recruits the nucleosome remodelling factor (NURF) complex to the SOX4 promoter, activating oncogenic transcription programs that increase gastric cancer cell malignancy [51]. These paradigms collectively demonstrate that circRNAs recruit specific activator complexes to target gene promoters and thereby modulate downstream oncogenic pathways.

#### Acting as protein scaffolds

Emerging evidence demonstrates the capacity of circRNAs to orchestrate protein complex assembly through ternary interactions. One pioneering study reported that circACC1 combines with the AMPK β/γ subunits to form a ternary complex, enhancing the stability and catalytic activity of the AMPK holoenzyme [52]. Another study indicated that circ-LRIG3 assembles a circ-LRIG3–EZH2–STAT3 ternary complex that facilitates EZH2-mediated STAT3 methylation and phosphorylation, eventually activating STAT3 signalling [53]. Additionally, Du et al. revealed that in noncancerous cells, circ-Foxo3 forms a ternary complex with CDK2 and p21 that blocks the function of CDK2, ultimately inducing cell cycle arrest and suppressing proliferation [54]. The above examples indicate that circRNAs function as molecular scaffolds to perform three critical functions: (1) enzymatic activity modulation, (2) protein complex stabilization, and (3) spatial coordination of protein‒protein interactions.

#### Interacting with specific proteins

Nuclear circPDIA4 competitively binds the RNA helicase DHX9, disrupting its interaction with target RNAs and thereby enhancing DHX9-dependent circRNA biogenesis and accelerating gastric cancer progression [55]. Moreover, circMTCL1 interacts with C1QBP protein and augments C1QBP translational output by inhibiting ubiquitin-proteasomal degradation [56]. In cervical cancer, circVPRBP overexpression strongly represses lymph node metastasis by interacting with RACK1 and shielding its S122 O-GlcNAcylation site to accelerate RACK1 degradation [57]. Taken together, these findings indicate that circRNAs can interact with specific proteins to change their routine biological functions or influence downstream biological processes.

#### Several controversies

With regard to the effect of circRNA-protein binding, the abundance of circRNAs should be considered, as was argued above for miRNA sponging mechanisms. In addition, RNA immunoprecipitation (RIP), RNA pull-down, and colocalization analyses to validate these interactions are essential. Notably, the secondary and tertiary structures of proteins and circRNAs may also affect their affinities, although this aspect has not been thoroughly examined in current research.

### Translating proteins or peptides

A pivotal breakthrough in 2017 emerged from three independent studies demonstrating m^6^A-dependent and IRES-mediated translation mechanisms in circRNAs [58–60]. A growing number of tumour-related circRNAs have subsequently been identified to possess protein-coding potential. For example, circZKSCAN1 encodes a 206-amino-acid polypeptide through an IRES-driven open reading frame (ORF) to promote the ubiquitination of mTOR, thereby inhibiting the PI3 K/AKT/mTOR pathway in hepatocellular carcinoma [61]. In addition, circARHGAP35 contains an ORF with an m^6^A-modified initiation codon that encodes a truncated protein that contributes to cancer progression [62]. Moreover, m^6^A modifications of circ-ZNF609, which was previously proven to have protein-coding ability owing to its IRES activity, can accelerate its IRES-mediated translation [63]. These discoveries establish two non-mutually exclusive translation initiation mechanisms, namely, m^6^A-dependent translation and IRES-mediated translation. These findings fundamentally expand our understanding of the mechanisms governing circRNA-derived proteins and polypeptides.

## CircRNA profiles in AML

The continuous evolution of RNA sequencing and microarray technologies has revolutionized transcriptome-level gene expression analysis in AML, enabling the systematic identification of functional circRNAs. Microarray assays and next-generation sequencing (NGS) are the most widely applied methods for circRNA research.

Notably, several novel technologies, such as single-cell RNA sequencing [64], Nanopore sequencing [65–67], and electrochemical detection [68, 69], have been applied to increase sequencing throughput, circRNA capture efficiency, and detection specificity.

To determine whether circRNAs are involved in AML progression, extensive studies have been conducted to compare circRNA expression between AML patients and healthy controls [70–77]. These studies consistently demonstrate widespread dysregulation of circRNAs in AML [70–77]. Notably, Ding et al. reported that differentially expressed circRNAs in AML are enriched in biological processes including cell proliferation, migration, and response to drugs and are closely associated with protein binding, ATP binding and RNA binding functions [74]. KEGG pathway analysis further revealed that these circRNAs are involved mainly in ErbB signalling, EGFR tyrosine kinase inhibitor resistance and mTOR signalling pathways, all of which are related to the development of AML [74]. Moreover, three other studies reported that AML-associated circRNAs are primarily exon-derived and show chromosomal distribution biases, with high frequencies on chromosomes 1, 2, 6, and 16 and minimal representation on chromosomes 13 and 21, and in the mitochondrial DNA [71, 72, 76]. These unbalanced distribution patterns suggest that circRNAs from chromosomes 1, 2, 6, and 16 may preferentially regulate AML progression. Growing evidence has revealed that circRNAs are strongly implicated in malignancy-related behaviours and treatment response [78–82]. Lv et al. identified 512 differentially expressed circRNAs (253 upregulated, 259 downregulated) between samples from AML with and without extramedullary infiltration (EMI) [78]. Through network analysis, these authors mapped a circRNA‒miRNA‒gene interaction atlas and pinpointed 17 circRNAs associated with migration, adhesion, signal transduction and cell‒cell communication, suggesting that they are likely responsible for EMI [78]. In addition, Li et al. discovered 1824 dysregulated circRNAs in adriamycin-resistant AML cells that were predominantly linked to B/T-cell receptor signalling, MAPK signalling, and mTOR signalling [81]. These results suggest that circRNAs play pivotal roles in the malignant progression of AML.

Gene mutations, including Nucleophosmin (NPM1), FMS-like tyrosine kinase 3 (FLT3), and splicing factor mutations (e.g., SF3B1, SRSF2, U2 AF1), are recognized as key drivers of AML pathogenesis [83, 84]. Notably, circRNA expression profiles are obviously influenced by genetic alterations [46, 84, 85]. For example, comparative analysis of haematopoietic stem/progenitor cells (HSPCs) from AML patients and healthy controls revealed 124 dysregulated circRNAs in patients with NPM1 mutations and 42 dysregulated circRNAs in patients with splicing factor mutations [84]. Two other studies have corroborated these findings [46, 85].

Emerging studies have demonstrated that m^6^A modifications are prevalent in numerous circRNAs and regulate 5’-cap-independent translation and selective degradation [37, 58–60]. However, the biological function of m^6^A-modified circRNAs remains poorly understood. To address this, Issah et al. conducted a circRNA epitranscriptomic microarray assay in AML, identifying 1136 differentially expressed m^6^A-modified circRNAs between AML patients and healthy controls [86]. Among these genes, 1057 were upregulated, and 79 were downregulated [86]. Subsequent GO annotation and pathway analysis implicated these circRNAs in AML tumorigenesis [86].

In this section, we summarize the circRNA profiling studies that have been performed in AML to date (Table 1). While circRNAs are broadly dysregulated in AML, only a limited fraction of them have been explored. We expect that our summary will serve as a valuable resource for further investigations.

**Table 1:** CircRNA profiles in AML

GEO DataSets	Samples	Method	Differential circRNAs	References
GSE94591	Healthy controls (n = 4) and newly diagnosed CN-AML patients (n = 6)	Arraystar Human CircRNA Array	464 circRNAs were differentially expressed, in which 147 circRNAs were upregulated and 317 circRNAs were downregulated	[70]
–	IDA samples (n = 5) and AML patients (n = 5)	Arraystar Human CircRNA Array	698 circRNAs were differentially expressed, among them, 282 were upregulated and 416 were downregulated	[71]
–	Healthy children (n = 6) and pediatric AML patients (n = 6)	Arraystar Human CircRNA Array	Of the 569 differentially expressed circRNAs, 273 were upregulated and 296 were downregulated	[72]
–	IDA controls (n = 3) and AML patients (n = 3)	Arraystar Human CircRNA Array	Numerous circRNAs were dysregulated in AML	[73]
–	Healthy controls (n = 5) and AML patients (n = 5)	Arraystar Human CircRNA Array	173 circRNAs were upregulated and 181 circRNAs were downregulated in AML	[74]
GSE116618	Normal individuals (n = 4) and AML patients (n = 8) based on GSE116617	Arraystar Human CircRNA Array	19 circRNAs were differentially expressed	[75]
–	Normal individuals (n = 5) and pediatric AML patients (n = 5)	Arraystar Human CircRNA Array	A total of 1960 circRNAs were differentially expressed with 1001 circRNAs found to be upregulated and 959 downregulated	[76]
GSE163386	Healthy controls (n = 4) and AML patients (n = 5)	Arraystar Human CircRNA Array	CircRNAs were abnormally expressed in AML	–
GSE94591, GSE163386	From the two datasets	Arraystar Human CircRNA Array	68 and 13 significantly upregulated circRNAs screened from the two datasets, respectively	[77]
GSE116617	Non-EMI AML patients (n = 4) and EMI AML patients (n = 4)	Arraystar Human CircRNA Array	253 circRNAs were upregulated and 259 circRNAs were downregulated in AML patients with EMI compared to those without EMI	[78]
–	NB4 cells with or without RNase R digestion upon ATRA treatment for 24 h and 48 h	Illumina HiSeq	508 circRNAs with dynamic expression during ATRA treatment, including 246 upregulated and 262 downregulated	[79]
–	THP-1/ADM (n = 3) and THP-1 (n = 3); K562/ADM (n = 3) and K562 (n = 3)	Illumina HiSeq	29 circRNAs were differentially expressed between the two cell groups, of which 18 were upregulated and 11 downregulated	[80]
–	HL-60/ADM cells (n = 3) and HL60 cells (n = 3)	Illumina HiSeq	A total of 1824 circRNAs were abnormally expressed in HL-60/ADM cells	[81]
–	Controls (n = 4), CR pediatric AML patients (n = 4), and non-CR pediatric AML patients (n = 4)	Arraystar Human CircRNA Array	378 upregulated and 688 downregulated circRNAs in pediatric AML patients vs. controls; 832 upregulated and 950 down-regulated circRNAs in CR AML patients vs. non-CR AML patients	[82]
GSE94591	FLT3-ITD^+^ (n = 3) and FLT3-ITD^−^ (n = 3) AML patients	Arraystar Human CircRNA Array	A total of 373 circRNAs were differentially expressed	[46]
GSE158596	Healthy HSPC (n = 16) and AML patients (n = 61, including 20 NPM1 mutated and 16 splicing factors mutated)	Illumina HiSeq	Compared with healthy HSPC, 124 circRNAs and 42 circRNAs were differentially expressed in NPM1 mutated AML and splicing factors mutated AML, respectively	[84]
–	NPM1 mutated AML (n = 5) and NPM1 wild-type AML (n = 5)	Illumina HiSeq	CircRNAs were aberrantly expressed	[85]
–	Healthy control (n = 3) and AML (n = 4)	Arraystar Human CircRNA Epitran-scriptomic Microarray	1136 m^6^A modified circRNAs were differentially expressed in the two groups, including 1057 up-regulated and 79 downregulated	[86]

*circRNA* circular RNA, *AML* acute myeloid leukaemia, *GEO* Gene Expression Omnibus, *CN-AML* cytogenetically normal acute myeloid leukaemia, *IDA* iron-deficiency anaemia, *EMI* extramedullary infiltration, *ATRA* all-trans retinoic acid, *ADM* Adriamycin (Doxorubicin), *CR* complete response, *vs* versus, *FLT3* FMS-like tyrosine kinase-3, *ITD* internal tandem duplication, *HSPC* haematopoietic stem and progenitor cell, *NPM1* nucleophosmin, *m*^*6*^*A* N6-methyladenosine

## Online databases and tools for circRNA exploration

Research on circRNAs is still at a preliminary stage, with their functions and regulatory networks remaining incompletely understood. To bridge this knowledge gap, various specialized databases and analytical tools that provide circRNA profiles and annotations, assess their protein-coding potential and predict their potential functions have been developed. In this section, we summarize open-access platforms for circRNA exploration (Tables 2, 3, 4), highlighting their unique capabilities and applications.

**Table 2:** Databases or tools relate to circRNA profiles, identification, and annotation

Name	Short description	Address	References
GEO	Helping users query and download experiments and curated gene expression profiles	https://www.ncbi.nlm.nih.gov/geo/	[87]
circRNADisease v2.0	Involving in 2964 circRNAs, 416 diseases and 9 species. Facilitating users to browse, search, and download circRNA-disease association data	http://cgga.org.cn:9091/circRNADisease/	[88]
deepBase v3.0	Displaying and analyzing of expression, evolution, function of various ncRNAs, including circRNAs	https://rna.sysu.edu.cn/deepbase3/	[89]
CircAtals 3.0	Integrating the most comprehensive circRNAs and their expression and functional profiles in vertebrates	https://ngdc.cncb.ac.cn/circatlas/	[90]
CircSC	Employing a compendium of full-length single-cell RNA-sequencing datasets and identifying 196,491 human and 310,969 mouse circRNAs	https://ngdc.cncb.ac.cn/circatlas/circSC/index.html	–
CSCD 2.0	Identifying significant number of circRNAs in human cancer and normal tissues/cell lines (~ 2.9 millions)	http://geneyun.net/CSCD2/	[91]
CircNet 2.0	Providing tissue-specific circRNA expression	https://awi.cuhk.edu.cn/~CircNet	[92]
exoRBase v2.0	Providing the comprehensive annotation and expression landscapes of extracellular vesicles long RNAs, which contain mRNA, lncRNA, and circRNA	http://www.exoRBase.org	[93]
MiOncoCirc	Provideing a compendium of circRNAs compiled from cancer clinical samples at The University of Michigan	https://mioncocirc.github.io/	[94]
Circ2Disease	Facilitating users to browse, search, and download circRNA-disease association data	http://bioinformatics.zju.edu.cn/Circ2Disease/index.html	[95]
TSCD	Providing a global view of tissue-specific circRNA in main tissues of human and mouse	http://gb.whu.edu.cn/TSCD	[96]
CircRic	Providing circRNAs expression profile in 935 cancer cell lines from CCLE	https://hanlab.uth.edu/cRic	[97]
CIRI	A tool for identifying de novo circRNAs in RNA-seq data	https://sourceforge.net/projects/ciri/	[28]
CIRCexplorer2	A tool for circRNAs identification and characterization from non-polyadenylated RNA-seq datasets	http://circexplorer2.readthedocs.org	[98]
circRNA_finder	A tool for identifying circRNAs in RNA-seq data	https://github.com/orzechoj/circRNA_finder	[99]
find_circ	A tool for identifying circRNAs in RNA-seq data	https://github.com/marvin-jens/find_circ	[100]
DCC	Applying several logical filters and integrating data across replicate sets to arrive at a precise list of circRNA candidates from sequencing data	https://github.com/dieterich-lab/DCC	[101]
NCLscan	Identifying non-co-linear transcripts with a good balance between sensitivity and precision from RNA-seq data	https://github.com/TreesLab/NCLscan	[102]
circBase	Facilitating users to browse and download gene annotations, and obtain expression details of a particular circRNA	http://www.circbase.org	[103]
CircBank	Including more than 140,000 human annotated circRNA from different source. Allowing users to query the circRNA information based on different search criteria	http://www.circbank.cn	[104]
circVAR	Providing resources for circRNA-related genetic variants in healthy and diseased populations	http://soft.bioinfo-minzhao.org/circvar	[105]
circRNADb	Containing 32,914 annotated exonic circRNAs	http://reprod.njmu.edu.cn/cgi-bin/circrnadb/	[106]

*circRNA* circular RNA, *GEO* Gene Expression Omnibus, *mRNA* messenger RNA, *lncRNA* long non-coding RNA, *CCLE* Cancer Cell Line Encyclopedia, *RNA-seq* RNA sequencing

**Table 3:** Databases or tools for predicting the translation potential of circRNA

Name	Short description	Address	References
CircAtals 3.0	Predicting potential ORFs and IRESs on circRNAs	https://ngdc.cncb.ac.cn/circatlas/	[90]
CSCD 2.0	Providing ORF sequence of circRNAs	http://geneyun.net/CSCD2/	[91]
CircNet 2.0	Providing ORF sequence of circRNAs	https://awi.cuhk.edu.cn/~CircNet	[92]
circRNADb	Predicting potential ORFs and IRESs on circRNAs	http://reprod.njmu.edu.cn/cgi-bin/circrnadb/	[106]
ORF finder	Searching for ORFs in the DNA sequence	https://www.ncbi.nlm.nih.gov/orffinder/	[107]
CircPro	Providing the coding/noncoding classification, the coding potential score and the information of ORF	http://bis.zju.edu.cn/CircPro	[108]
IRESfinder	A python package for identifying RNA IRESs in eukaryotic cell	https://github.com/xiaofengsong/IRESfinder	[109]
IRESbase	Providing experimentally validated IRESs in the published literature, including mRNAs, circRNAs, and lncRNAs	http://reprod.njmu.edu.cn/cgi-bin/iresbase/index.php	[110]
CircCode	A Python3-base pipeline for identifying translatable circRNAs in ribo-seq reads	https://github.com/PSSUN/CircCode	[111]
Circbank	Searching for circRNA protein coding potential and circRNA m^6^A modification	http://www.circbank.cn	[104]
TransCirc	Providing evidences of circRNA translation, including ribosome/polysome profiling, translation initiation site, IRES sequence, m^6^A sites, ORF length, sequence composition, and proteomics evidence by Mass spectrometry	https://www.biosino.org/transcirc	[112]
CircPrimer 2.0	Predicting ORFs, IRESs and m^6^A sites	http://www.bioinf.com.cn/	[113]
SRAMP3	Predicting m^6^A modification sites on the RNA sequences of interests	http://www.cuilab.cn/sramp	[114]

*circRNA* circular RNA, *ORFs* open reading frame, *IRES* internal ribosome entry site, *DNA* deoxyribonucleic acid, *mRNA* messenger RNA, *lncRNA* long non-coding RNA, *ribo-seq* ribosome profiling

**Table 4:** Databases for predicting the potential regulatory mechanisms of circRNA

Name	Short description	Address	References
CSCD 2.0	Predicting the interactions of circRNA-miRNA and circRNA-RBP	http://geneyun.net/CSCD2/	[91]
CircNet 2.0	Providing circRNA-miRNA-gene regulatory network	https://awi.cuhk.edu.cn/~CircNet	[92]
Circ2Disease	Providing circRNA-miRNA-gene regulatory networks in human diseases	http://bioinformatics.zju.edu.cn/Circ2Disease/index.html	[95]
CircRic	Analyzing the regulators in circRNAs biogenesis and effect of circRNAs on drug response. Providing the association between circRNAs with mRNA, protein and mutation. Predicting RBP and miRNA binding site in circRNAs	https://hanlab.uth.edu/cRic	[97]
Circbank	Providing miRNA-circRNA interactions and circRNA mutation information	http://www.circbank.cn	[104]
CircInteractome	Predicting the binding sites for RBPs and miRNAs on reported circRNAs	http://circinteractome.nia.nih.gov	[115]
CircFunBase	Providing visualized circRNA-miRNA and circRNA-RBPs interaction networks and function information of circRNAs	http://bis.zju.edu.cn/CircFunBase	[116]
ENCORI	Providing RBP-circRNA interactions which are supported by CLIP-seq data	https://rnasysu.com/encori/index.php	[117]
TRCirc	Integrating current transcription factors binding sites and circRNA annotations. Also, providing other information of circRNAs,such as methylation level, H3 K27ac signals, enhancers and expression	http://www.licpathway.net/TRCirc	[118]

*circRNA* circular RNA, *miRNA* microRNA, *RBP* RNA binding protein, *mRNA* messenger RNA, *CLIP-seq* chromatin immunoprecipitation followed by sequencing, *H3 K27ac* histone H3 lysine 27 acetylation

There are already several databases containing circRNA profiles associated with various diseases, such as GEO [87], circRNADisease v2.0 [88], deepBase v3.0 [89], CircAtlas 3.0 [90], CircSC, CSCD 2.0 [91], CircNet 2.0 [92], exoRBase v2.0 [93], MiOncoCirc [94], Circ2Disease [95], TSCD [96], and CircRic [97]. These databases help users browse, search, and download information related to circRNAs. Notably, CircAtlas also provides an ID conversion service that can convert IDs from different circRNA databases [90]. In addition, circSC integrates a substantial number of full-length single-cell RNA-sequencing datasets, including a total of 196,491 human and 310,969 mouse circRNAs, and provides information on the specific expression patterns of circRNAs in different cells and samples. CircRic provides circRNA expression profiles in 935 cancer cell lines across 22 cancer lineages from the Cancer Cell Line Encyclopedia (CCLE) [97]. CIRI [28], CIRCexplorer2 [98], circRNA_finder [99], find_circ [100], DCC [101], and NCLscan [102] are commonly used tools for identifying circRNAs in large-scale RNA sequencing data. Additionally, circBase [103], CircBank [104], circVAR [105], and circRNADb [106] contain detailed annotations of circRNAs. Notably, the circVAR database provides resources for identifying circRNA-related genetic variants in healthy and diseased populations and allows users to quickly search for genetic variants in circRNAs and download all annotated variants [105].

CircRNAs can serve as miRNA sponges, interact with DNA or proteins, and encode proteins. The most likely mode of action can be predicted via bioinformatics tools. For example, CircAtlas 3.0 [90], CSCD 2.0 [91], CircNet 2.0 [92], circRNADb [106], ORF-FINDER [107], CircPro [108], IRESfinder [109], and IRESbase [110] can predict the protein-coding potential of circRNAs on the basis of the ORFs and/or IRESs present. CircCode, a Python 3-based framework for recognizing translatable circRNAs in ribo-seq reads, is also a powerful and convenient research tool [111]. Recent studies have demonstrated that m^6^A modification of circRNAs plays important roles in driving translation and mediating degradation [37, 58–60]. Therefore, predicting m^6^A modification sites in circRNAs is also essential. CircBank [104], TransCirc [112], and CircPrimer 2.0 [113] provide evidence of circRNA features related to translation, including ORFs, IRESs, and m^6^A modifications. Notably, in addition to the above services, TransCirc can perform integrative analysis to predict the coding potential of a circRNA and provides ribosome/polysome profiling, translation initiation site, sequence composition, and proteomics evidence from mass spectrometry while allowing users to search by circRNA sequence [112]. CircPrimer 2.0 can help researchers design primers for circRNAs and determine their specificity [113]. In addition, SRAMP3 serves as a useful tool to predict m^6^A modification sites in RNA sequences of interest [114]. Regarding other mechanisms of circRNA functions, some databases provide guidance for future research. For example, CSCD 2.0 [91], CircNet 2.0 [92], Circ2Disease [95], CircRic [97], CircBank [104], CircInteractome [115], CircFunBase [116], ENCORI [117], and TRCirc [118] provide interactions between circRNAs and miRNAs or RBPs. Interestingly, CircRic also provides information related to the regulators of circRNA biogenesis, the effect of circRNAs on drug response, and the associations between circRNAs and mutations [97]. CircBank also provides circRNA mutation information [104]. In addition, CircInteractome can be used to design divergent primers and siRNAs for circRNAs [115]. Notably, TRCirc not only contains information related to the transcriptional regulation of circRNAs but also provides related information such as data on enhancers, methylation, H3 K27ac modification and circRNA expression [118].

In conclusion, these databases and tools provide convenient platforms that allow researchers to explore the expression atlases, basic information and potential functions of circRNAs. However, predictions derived from databases require experimental validation. In addition, the lack of standardized nomenclature for circRNAs complicates database searches. Moreover, in contrast to the case for mRNAs, miRNAs, and lncRNAs, clinical databases specifically dedicated to circRNAs remain underdeveloped. Furthermore, current databases lack information on circR loops.

## Roles of circRNAs in AML

**Fig. 2:**
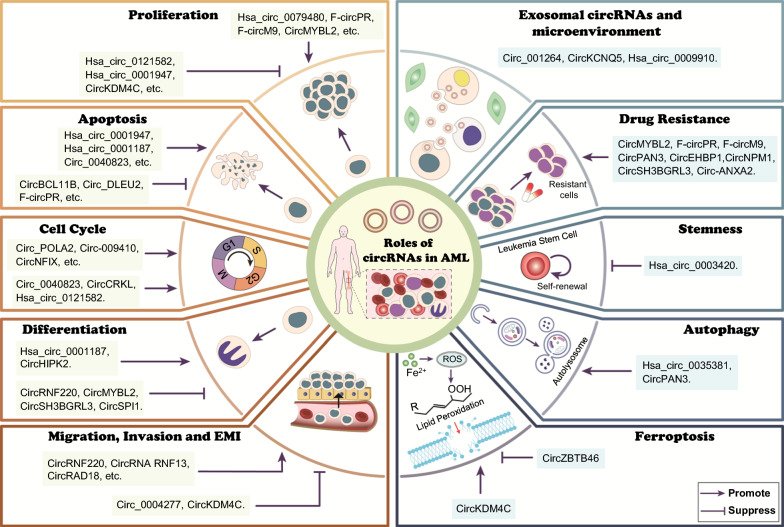
The biological roles of circRNAs in AML. CircRNAs regulate cell proliferation, apoptosis, the cell cycle, differentiation, migration, invasion, extramedullary infiltration, ferroptosis, autophagy, stemness, drug resistance, exosomes and the tumour microenvironment in AML

Recent studies have demonstrated that circRNAs play pivotal roles in the initiation and progression of haematological malignancies, especially AML. In the following section, we describe the biological roles of circRNAs in cell proliferation, apoptosis, the cell cycle, differentiation, migration, invasion, extramedullary infiltration, ferroptosis, autophagy, stemness, drug resistance, exosomes and the tumour microenvironment in AML (Table 5, Fig. 2).

**Table 5:** Overview of dysregulated circRNAs in AML

CircRNAs	Host gene	Level	Mechanism	Downstream molecule/signaling	Biological Function	References
CircMYBL2 (Hsa_circ_0006332)	MYBL2	↑ in FLT3-ITD^+^ AML	Bind to PTBP1	FLT3, STAT5, c-MYC, MCL1, p27/Kip1	Proliferation (+); Apoptosis (−); Cell cycle progress (+); Differentiation (−); Drug resistance (+)	[46]
Hsa_circ_0004136 (Circ_KCNQ5)	KCNQ5	↑ in AML; ↑ in the exosomes derived from AML serum and cell lines	Sponge miR-142; Sponge miR-622 and derepress RAB10; Sponge miR-570-3p and derepress TSPAN3	PCNA	Proliferation (+); Apoptosis (−); Cell cycle progress (+); Migration and invasion (+)	[72, 140, 141]
Hsa_circ_0009910	MFN2	↑ in AML; ↑ in the exosomes derived from AML cell lines	Sponge miR-20a-5p; Sponge miR-5195-3p and derepress GRB10	Bax, Bcl-2	Proliferation (+); Apoptosis (−); Cell cycle progress (+)	[73, 148]
Circ-ANXA2 (Hsa_circ_0035559)	ANXA2	↑ in AML	Target miR-23a-5p and miR-503-3p	NR	Proliferation (+); Apoptosis (−); Drug resistance (+)	[74]
CircTASP1 (Hsa_circ_406083; Hsa_circ_0007340)	TASP1	↑ in AML	Sponge miR-515-5p and upregulate HMGA2	Bax, Cleaved caspase-3, Bcl-2	Proliferation (+); Apoptosis (−); Cell cycle progress (+)	[75]
CircRNF220 (Hsa_circ_0012152)	RNF220	↑ in AML; ↑ in AML serum	Through miR-30a/MYSM1, miR-30a/IER2; miR-625-5p/SOX12; miR-330-5p/SOX4 axis	CyclinD1, Cleaved caspase-3; NF-κB, mTOR, FoxO signaling	Proliferation (+); Apoptosis (−); Cell cycle progress (+); Differentiation (−); Migration and invasion (+)	[76, 134, 135]
CircZBTB46 (Hsa_circ_103104)	ZBTB46	↑ in AML	Sponge miR-671-5p and derepress SCD	PI3 K-AKT, MAPK, mTOR signaling	Proliferation (+); Cell cycle progress (+); Ferroptosis (−)	[77]
CircBCL11B	BCL11B	↑ in AML	NR	NR	Proliferation (+); Apoptosis (−)	[84]
Hsa_circ_0079480	ISPD	↑ in AML	Sponge miR-654-3p and derepress HDGF	NR	Proliferation (+); Apoptosis (−)	[119]
F-circPR	PML-RARα	de novo in AML	NR	AKT signaling	Proliferation (+); Apoptosis (−); Drug resistance (+)	[120]
F-circM9	MLL-AF9	de novo in AML	NR	MAPK, AKT signaling	Proliferation (+); Apoptosis (−); Drug resistance (+)	[121]
Circ-PTK2 (Hsa_circ_104700; Hsa_circ_0005273)	PTK2	↑ in AML	Sponge miR-330-5p and upregulate FOXM1	CyclinD1, Bcl-2, Bax	Proliferation (+); Apoptosis (−)	[122]
Circ_POLA2	POLA2	↑ in AML	Target mature miR-34a and upregulate PD-L1	CDK4, CDK6	Proliferation (+); Cell cycle progress (+)	[123]
Circ_DLEU2 (Hsa_circ_0000488)	DLEU2	↑ in AML	Sponge miR-582-5p and upregulate COX2; Sponge miR-496 and upregulate PRKACB	Ki67, Bcl-2, Bax	Proliferation (+); Apoptosis (−); Cell cycle progress (+)	[124]
Circ_0094100	NR	↑ in AML	Sponge miR-217 and derepress ATP1B1	PCNA, CyclinD1, Bcl-2	Proliferation (+); Apoptosis (−); Cell cycle progress (+)	[125]
CircNFIX	NFIX	↑ in AML	Sponge miR-876-3p and derepress TRIM31	CyclinD1, Bcl-2, Cleaved caspase-3	Proliferation (+); Apoptosis (−); Cell cycle progress (+)	[126]
Hsa_circ_0000370	FLI-1	↑ inAML; ↑ in FLT3-ITD^+^ AML	Sponge miR-1299 and upregulate S100 A7 A	NR	Proliferation (+); Apoptosis (−); Cell cycle progress (+)	[127]
Circ_0104700	HOMER2	↑ in AML	Sponge miR-665 and derepress MCM2	JAK/STAT signaling	Proliferation (+); Apoptosis (−); Cell cycle progress (+)	[128]
Hsa_circ_0044907	RPS6 KB1	↑ in AML	Sponge miR-186-5p and derepress KIT	PCNA, CyclinD1, Cleaved caspase-3	Proliferation (+); Apoptosis (−); Cell cycle progress (+)	[129]
Hsa_circ_0002483	PTK2	↑ in AML	Sponge miR-758-3p and derepress MYC	Cleaved caspase-3, caspase-3, Bax, Bcl-2	Proliferation (+); Apoptosis (−); Cell cycle progress (+)	[130]
Hsa_circ_ 0005774	CDK1	↑ in AML	Sponge miR-192-5p and upregulate ULK1	PCNA, CyclinD1, Bcl-2	Proliferation (+); Apoptosis (−); Cell cycle progress (+)	[131]
CircSPI1	SPI1	↑ in AML	Sponge miR-1307-3p, miR-382-5p, miR-767-5p; Interact with eIF4 AIII	PU.1, p-ERK1/2, Bcl-2, CDK6; Ras, PI3 K/AKT, p53, JAK-STAT, FoxO signaling	Proliferation (+); Apoptosis (−); Differentiation (−)	[132]
CircSH3BGRL3	SH3BGRL3	↑ in AML	Sponge miR-375-3p and derepress YAP1	NR	Proliferation (+); Cell cycle progress (+); Differentiation (−); Drug resistance (+)	[133]
CircRNF13 (Hsa_circ_0001346)	RNF13	↑ in AML	Sponge miR-1224-5p	c-MYC, caspase-3/7, Tenascin-C	Proliferation (+); Apoptosis (−); Cell cycle progress (+); Migration and invasion (+)	[136]
CircRAD18	RAD18	↑ in AML	Sponge miR-206 and derepress PRKACB	Bax, Bcl-2, Cleaved caspase-3	Proliferation (+); Apoptosis (−); Cell cycle progress (+); Migration and invasion (+)	[137]
CircPLXNB2 (Hsa_circ_0001257)	PLXNB2	↑ in AML	Sponge miR-654-3p and derepress CCND1	PLXNB2, CyclinD1, Bax, PCNA, Bcl-2	Proliferation (+); Apoptosis (−); Cell cycle progress (+); Migration and invasion (+)	[138, 139]
Hsa_circ_0013880	TXNIP	↑ in AML	Target USP32/Rap1b axis	CyclinD1, Cleaved caspase-3/9, Cleaved-PARP, CDK4, p21	Proliferation (+); Apoptosis (−); Cell cycle progress (+); Migration and invasion (+)	[142]
Circ-SFMBT2 (Hsa_circ_0017639)	SFMBT2	↑ in AML; ↑ in AML serum	Sponge miR-582-3p and upregulate ZBTB20	CyclinD1, MMP9, Bax	Proliferation (+); Apoptosis (−); Migration and invasion (+)	[143]
Hsa_circ_0003602 (Circ-SMARCC1)	SMARCC1	↑ in AML	Sponge miR-502-5p and upregulate IGF1R	NR	Proliferation (+); Apoptosis (−); Migration and invasion (+)	[144]
Circ_0058058	NR	↑ in AML	Sponge miR-4319 and upregulate EIF5 A2	CyclinD1, Bcl-2, Cleaved caspase-3	Proliferation (+); Apoptosis (−); Migration and invasion (+)	[145]
CircPVT1	PVT1	↑ in AML	Stabilize c-Myc protein	CXCR4	Proliferation (+); Apoptosis (−); Migration and invasion (+)	[146]
CircNPM1 (Circ_0075001)	NPM1	↑ in AML; ↑ in AML serum	Sponge miR-345-5p and derepress FZD5	NR	Proliferation (+); Apoptosis (−); Cell cycle progress (+); Migration and invasion (+); Drug resistance (+)	[147]
Hsa_circ_0035381	PIGB	↑ in AML	Sponge miR-582-3p and derepress YWHAZ	LC3-II/I, Beclin1	Proliferation (+); Apoptosis (−); Cell cycle progress (+); Autophagy (+); Oxidative stress (−)	[149]
Circ_001264	ST6GALNAC3	↑ in AML; ↑ in AML cell-derived exosomes	Sponge miR-502-5p and derepress RAF1	p38-STAT3	Proliferation (+); Apoptosis (−); M2 polarization of macrophages (+)	[150]
Hsa_circ_0121582	GSK3β	↓ in AML	Sponge miR-224 and derepress GSK3β; Interact with TET1	Wnt/β-catenin signaling	Proliferation (−); Cell cycle progress (−)	[151]
Hsa_circ_0001947	AFF2	↓ in AML	Sponge miR-329-5p and upregulate CREBRF	NR	Proliferation (−); Apoptosis (+)	[152]
CircCRKL	CRKL	↓ in AML	Through miR-196a-5p/miR-196b-5p/p27 axis	NR	Proliferation (−); Cell cycle progress (−)	[153]
Circ_0040823	BANP	↓ in AML; ↓ in AML serum	Sponge miR-516b and derepress PTEN	CyclinD1, CyclinE1, Bcl-2, Bax, Cleaved caspase-3	Proliferation (−); Apoptosis (+); Cell cycle progress (−)	[154]
Circ_0004277	WDR37	↓ in AML	Sponge miR-134-5p and derepress SSBP2	NR	Proliferation (−); Migration and invasion (−)	[155]
Hsa_circ_0001187	DOPEY2	↓ in AML	Sponge miR-499a-5p and upregulate RNF113 A	METTL3, MDM2, p53, p21	Proliferation (−); Apoptosis (+); Differentiation (+)	[156]
CircKDM4 C	KDM4 C	↓ in AML	Sponge hsa-let-7b-5p and upregulate p53	ACSL4, PTGS2, GPX4, FTH1	Proliferation (−); Migration and invasion (−); Ferroptosis (+)	[157]
Circ-HIPK2	HIPK2	↓ in ATRA-treated NB4 cells	Sponge miR-124-3p	NR	ATRA-induced differentiation (+)	[79]
Hsa_circ_0003420	NR	↓ in non-M3 AML stem cells	Target IGF2BP1	Cleaved caspase-3, Bax, Bcl-2, HOXB4, MYB, ALDH1 A1	Stemness (−)	[165]
CircPAN3	PAN3	↑ in ADM-resistant AML cells	Through the regulation of autophagy; miR-153-5p/miR-183-5p-XIAP axis	LC3-II/I, Beclin1, p62, Cleaved caspase-3/9; AMPK/mTOR signaling	Autophagy (+); Drug resistance (+)	[80, 166]
CircEHBP1	EHBP1	↑ in AML; ↑ in ADR-resistant AML cells	Suppress miR-129 maturation	NR	Drug resistance (+)	[167]

↑ and ↓ indicate upregulation and downregulation respectively. + and − stand for facilitatory and inhibitory effects respectively. *circRNA* circular RNA, *AML* acute myeloid leukaemia, *ATRA* all-trans retinoic acid, *ADM* Doxorubicin, *ADR* Adriamycin, *NR* not report

### CircRNAs affect AML cell proliferation and apoptosis

Cancer cells, including AML, need to evade apoptosis in order to continue proliferating. Accumulating evidence indicates that circRNAs are highly involved in regulating cell proliferation and apoptosis. Specifically, some circRNAs, such as hsa_circ_0079480, f-circPR, and f-circM9, promote AML cell proliferation and suppress apoptosis [46, 72–77, 84, 119–150], whereas others, such as hsa_circ_0121582, hsa_circ_0001947, and hsa_circ_0003420, play diametrically opposite roles [151–157]. A detailed description of each circRNA is shown in Table 5. Mechanistically, circRNAs mainly exert their effects via circRNA–miRNA–mRNA networks, and few act by binding with proteins. For example, hsa_circ_0079480 can modulate AML proliferation and apoptosis through the miR-654-3p/HDGF axis [119]. Nuclear hsa_circ_0121582 can bind to the GSK3β promoter and recruit the DNA demethylase TET1 to GSK3β, thus potentiating the transcription of GSK3β and eventually suppressing cell proliferation [151]. Moreover, f-circM9 and f-circPR, which are derived from the MLL/AF9 and PML/RARα fusion genes, respectively, can also regulate AML cell proliferation and apoptosis, but the underlying mechanism remains unclear [120]. In addition, Conn et al. demonstrated that circR loops could promote AML initiation and progression both in vitro and in vivo through transcriptional pausing, proteasome inhibition, chromatin reorganization, and DNA breakage [44]. Thus, the functions of circRNAs in AML cell proliferation and apoptosis are becoming increasingly clear in the modern era of molecular biology.

### Cell cycle-associated circRNAs in AML

Cell cycle acceleration is a common feature of AML, leading to uncontrolled cell division and rapid proliferation [158]. Emerging evidence suggests that circRNAs are deeply involved in regulating the cell cycle progression of AML cells, leading to cell cycle acceleration or arrest [46, 73, 75–77, 122, 123, 125–131, 133–139, 141, 142, 147–149, 151, 153, 154]. Usually, circRNAs affect cell cycle progression by controlling cell cycle regulators such as cyclins, cyclin-dependent kinases (CDKs), and proliferating cell nuclear antigen (PCNA). For example, circ_POLA2 silencing causes the downregulation of CDK4 and CDK6, leading to G1/G0 cell cycle arrest in AML cells [122]. Circ_0094100 deficiency suppresses the protein levels of cyclin D1 and PCNA in rapamycin-treated AML cells and restrains the cell cycle [125]. Circ_0040823 sponges miR-516b to relieve the inhibitory effects of PTEN in AML, thereby inhibiting the expression of cell cycle-related proteins such as cyclin D1 and cyclin E1 and increasing the percentage of cells in the G0/G1 phase [154]. These results indicate that circRNAs play prominent roles in cell cycle regulation and that targeting circRNAs that function as negative regulators of the cell cycle may be a useful therapeutic strategy for AML.

### Cellular differentiation-related circRNAs in AML

Terminal differentiation block is one of the hallmarks of AML and results in the production of immature cells, ultimately leading to severe anaemia, infection, and bleeding [159]. Studies have shown that circRNAs participate in regulating AML differentiation. For example, circMYBL2, circSPI1, circSH3BGRL3 and circRNF220 are considered suppressors of differentiation in AML [46, 76, 132, 133]. In contrast, circ_0001187 and circ-HIPK2 promote AML cell differentiation [79, 156]. Mechanistically, most circRNAs function by sponging miRNAs. Due to the nonselective cytotoxicity of chemical agents towards both malignant and normal cells, differentiation-inducing therapy has emerged as a novel approach for treating AML with improved safety and efficacy. Therefore, circRNAs can serve as new targets for anticancer therapy in AML.

### CircRNAs modulate invasion, migration, and extramedullary infiltration in AML

Invasion, migration, and extramedullary infiltration (EMI) are malignant behaviours that often result in a high mortality rate and poor prognosis in AML patients. CircRNAs play key regulatory roles in cell invasion and migration, and the majority of related circRNAs, such as circRNF220, circRNF13, and CircRAD18, act by sponging miRNAs to interfere with important regulators of the abovementioned processes [135–138, 141–147, 155, 157]. EMI is associated with poor prognosis in AML patients owing to the associated destruction of vital organs. To further understand the expression profiles of circRNAs in AML with EMI, Lv et al. performed circRNA microarray analysis, including the construction of a circRNA‒miRNA‒mRNA regulatory network [78]. Seventeen circRNAs were identified as closely associated with EMI, but the exact mechanisms of the functions of those circRNAs remain unknown and require further investigation [78].

### CircRNAs regulate ferroptosis in AML

Ferroptosis is a recently identified type of cell death caused by iron-dependent lipid peroxidation, and targeting ferroptosis provides a new and promising approach for antitumour therapies [160]. In AML, Long et al. reported that circZBTB46 enhances the expression of stearoyl-CoA desaturase 1 (SCD), likely by acting as a miRNA sponge, thereby protecting AML cells from ferroptosis and promoting cell proliferation [77]. Moreover, circKDM4 C was reported to promote ferroptosis via the hsa-let-7b-5p/P53 axis [157]. Nevertheless, ferroptosis-related circRNAs have rarely been reported in AML and still require further characterization.

### Autophagy-related circRNAs in AML

Autophagy, an intracellular lysosome-dependent catabolic pathway, promotes tumour cell survival in response to multiple antitumour drugs, while sustained activation of autophagy can cause cell death [161, 162]. Shang et al. demonstrated that circPAN3 might facilitate AML resistance to doxorubicin through activating autophagy and the AMPK/mTOR signalling pathway [80]. Another study revealed that hsa_circ_0035381 deficiency could reduce autophagy levels and inhibit AML cell proliferation by regulating the miR-582-3p/YWHAZ axis in AML [149]. Currently, autophagy is considered a double-edged sword in tumours that can either promote cell survival or enable apoptosis [163]. However, reports on autophagy-related circRNAs in AML are scarce, and additional relevant circRNAs need to be identified. Targeting autophagy-related circRNAs may provide new strategies for AML treatment, especially for patients with drug-resistant disease.

### CircRNAs affect the stemness of leukaemia stem cells in AML

Cancer stem cells (CSCs) are considered the main cells responsible for tumour initiation, development, recurrence, metastasis and radiotherapy and chemotherapy failure [164]. Research by Lin et al. revealed that hsa_circ_0003420 was expressed at lower levels in non-M3 AML stem cells than in normal haematopoietic stem cells [165]. Its overexpression impaired the stemness of leukaemia stem cells and inhibited the expression of stemness‐related genes (HOXB4, MYB, ALDH1 A1, ABCB1, CD34, and MMRN1) [165]. However, there is a lack of related research in AML, and more studies are needed.

### CircRNAs regulate drug resistance in AML

To date, both conventional chemotherapeutic agents (e.g., Cytarabine, Adriamycin, and Daunorubicin) and novel targeted drugs (e.g., Venetoclax, Quizartinib, and Ivosidenib) for the treatment of AML have encountered drug resistance as an obstacle. Therefore, a better understanding of drug resistance mechanisms is vital for improving patient outcomes. Interestingly, numerous studies have demonstrated that circRNAs are involved in the regulation of drug resistance in AML. Two of those studies revealed that circPAN3 contributed to resistance to Adriamycin (Doxorubicin) through the regulation of autophagy and through the miR-153-5p/miR-183-5p-XIAP axis [80, 166]. In addition, circNPM1 and circEHBP1 were reported to be dysregulated in AML and involved in Adriamycin resistance by sponging miR-345-5p and miR-129, respectively [147, 167]. In addition, circMYBL2 induces resistance to quizartinib, a potent and highly selective FLT3 inhibitor, in FLT3-ITD^+^ AML cells by activating FLT3 kinase-dependent signalling pathways [46]. F-circM9 confers resistance to arsenic trioxide and cytarabine in AML [120]. Circ-ANXA2 and circSH3BGRL3 increase the chemosensitivity of AML cells to cytarabine and/or daunorubicin by regulating their target miRNAs [74, 133]. These results indicate that circRNAs play essential roles in drug resistance and that targeting circRNAs may be a promising treatment strategy for preventing drug resistance.

### Exosomal circRNAs and the AML microenvironment

The constant crosstalk between AML cells and their microenvironment affects tumour initiation and progression [168]. Exosomes are membranous vesicles secreted by virtually every type of living cell with an average diameter of ~ 100 nm. These vesicles are crucial executors of intercellular signalling and are also closely connected with the malignant behaviour of tumours [169, 170]. Notably, exosomal circ_001264, hsa_circ_0009910, and circ_0004136 are expressed at high levels in AML and play oncogenic roles in modulating AML cell behaviour [141, 148, 150]. Among them, exosomal circ_001264 can activate p38-STAT3 signalling to induce M2 macrophage polarization, thereby upregulating PD-L1 expression [150]. In addition, exosomal circ_001264 siRNA has been shown to inhibit AML tumorigenicity. PD-L1, a PD-1 ligand, interacts with PD-1 on the T-cell surface to attenuate T-cell activation and facilitate immune escape [171]. The coadministration of exosomal circ_001264 siRNA, anti-PD-L1 therapy, and cytarabine obviously increases antitumour activity in AML mouse models [150]. However, the mechanisms through which exosomal hsa_circ_0009910 and circ_0004136 function in the AML microenvironment still need to be explored [141, 148]. Taken together, these findings suggest that exosomal circRNAs play essential roles in regulating the malignant behaviours of tumour cells and cell-to-cell communication within the microenvironment and that interfering with circRNA expression may be an effective anticancer strategy. However, only a few exosomal circRNAs have been identified in AML, and their functions remain to be investigated further.

## Clinical application of circRNAs in AML

**Fig. 3:**
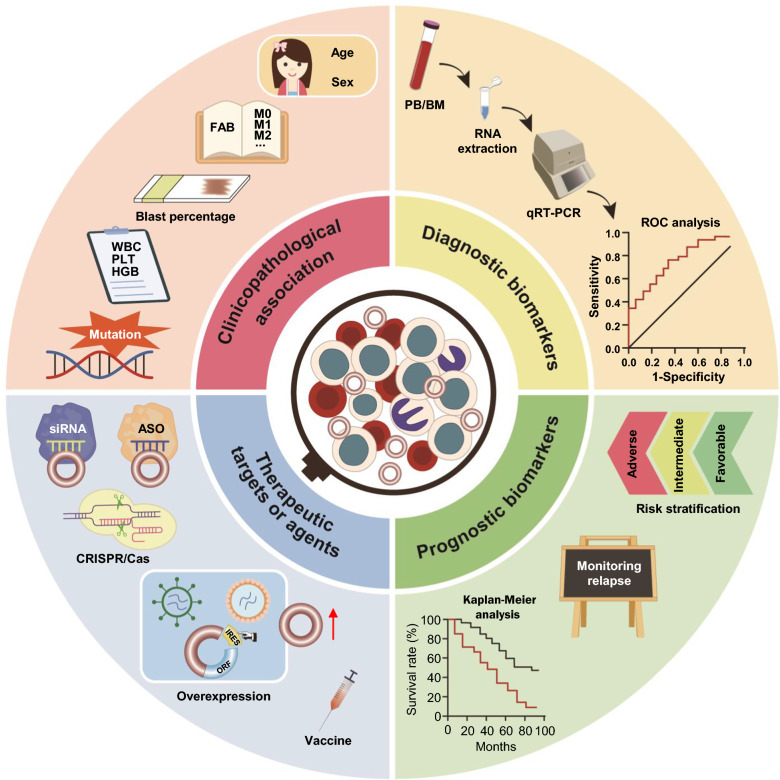
Clinical application of circRNAs in AML. CircRNAs are closely related to clinicopathologic features and have great potential as effective biomarkers for diagnosis and prognosis, as well as therapeutic targets in AML

Early diagnosis and timely treatment are highly important for improving cure rate and prognosis in patients with tumours. However, the current methods for the clinical diagnosis of tumours, such as tissue biopsy, endoscopy examination, and MRI, are often invasive, expensive, and time-consuming. The development of simple, minimally invasive, and relatively inexpensive approaches is essential to support early diagnosis. Moreover, the early identification of poor prognostic factors and timely delivery of targeted therapeutic interventions also improve clinical outcomes. Recent studies have demonstrated that certain circRNAs are closely associated with clinicopathologic features and possess great potential as effective biomarkers for diagnosis and prognosis, as well as therapeutic targets in AML (Fig. 3).

### Correlations between circRNA expression and clinicopathological characteristics in AML patients

In this section, we summarize the correlations between circRNA expression and clinicopathological characteristics in patients with AML according to the findings of recent studies (Table 6). For example, circ_0001187 is significantly decreased in older AML patients (aged > 43 years) [156]. AML patients with high circSMC1 A expression are more likely to be female [172]. CircEHBP1 is closely associated with French–American–British (FAB) classification [167]. Moreover, the expression levels of hsa_circ_001264, hsa_circ_0001947, circ_0001187, and circKLHL8 are inversely related to the percentage of blasts in the bone marrow (BM) or peripheral blood (PB), whereas circ-ANAPC7 and circ-PVT1 are positively related [150, 152, 156, 172–174]. High expression of hsa_circ_0001947, circ_0001187, and circFCHO2 and low expression of circ-ANAPC7 and hsa_circ_0079480 are correlated with a low white blood cell (WBC) count [152, 156, 172, 173, 175]. Circ_0001187 has a significant positive association with the platelet (PLT) count [156]. The levels of circTASP1, hsa_circ_0001947, and circ_0001187 are positively related to haemoglobin (HGB) levels [75, 152, 156]. Gene mutations are crucial events in AML pathogenesis [83]. Accumulating evidence suggests that circSMC1 A, circKLHL8, circFCHO2, circCFLAR, and circ-PVT1 are closely linked to mutations in genes such as NPM1, FLT3-ITD, WT1, or CEBPA [172, 174]. Overall, circRNA expression levels are strongly associated with clinicopathological features in AML patients.

**Table 6:** Correlation between circRNAs and clinicopathological characteristics of AML

Clinicopathologic features	Significantly associated circRNAs (P < 0.05)	References
Age	Circ_0001187	[156]
Sex: female	CircSMC1 A	[172]
FAB subtype	CircEHBP1	[167]
Percentages of blasts in BM	Circ_001264 Hsa_circ_0001947 Circ_0001187 Circ-ANAPC7 Circ-PVT1	[150] [152] [156] [173] [174]
Percentages of blasts in PB	Hsa_circ_0001947 Circ_0001187 CircKLHL8	[152] [156] [172]
WBC count	Hsa_circ_0001947 Circ_0001187 CircFCHO2 Circ-ANAPC7 Hsa_circ_0079480	[152] [156] [172] [173] [175]
PLT count	Circ_0001187	[156]
HGB level	CircTASP1 Hsa_circ_0001947 Circ_0001187	[75] [152] [156]
NPM1 mutation	CircFCHO2	[172]
FLT3-ITD	CircKLHL8 CircSMC1 A CircCFLAR CircFCHO2 Circ-PVT1	[172] [172] [172] [172] [174]
WT1 mutation	CircCFLAR	[172]
CEBPA Double mutation	CircFCHO2	[172]

*circRNA* circular RNA, *AML* acute myeloid leukaemia, *FAB* French-American-British, *BM* bone marrow, *PB* peripheral blood, *WBC* white blood cell, *PLT* platelet, *HGB* haemoglobin, *NPM1* nucleophosmin, *FLT3* FMS-like tyrosine kinase-3, *ITD* internal tandem duplication, *CEBPA* CCAAT Enhancer Binding Protein A, *WT1* Wilms’ Tumor 1

### CircRNAs as diagnostic biomarkers in AML

Several circRNAs have been reported to have diagnostic value in AML (Table 7). For example, Lin et al. revealed that circPLXNB2 was obviously elevated in BM samples from patients with AML and was valuable for distinguishing AML patients from healthy individuals (AUC = 0.8525) [138]. Other circRNAs in BM can also serve as diagnostic biomarkers, such as hsa_circ_0004277 (AUC = 0.957), circ-ANXA2 (AUC = 0.832), hsa_circ_0044907 (AUC = 0.9447), circ-ANAPC7 (AUC = 0.915), and circ_0059706 [70, 74, 77, 129, 152, 173, 174, 176, 177]. Although BM aspiration and biopsy are still the gold standard for diagnosing AML, these tests are invasive and cause physical trauma to patients. Moreover, repeated sampling is necessary during the course of treatment, leading to recurrent trauma. In contrast, PB collection and analysis is simpler, more cost-effective and less invasive. Studies have shown that circZBTB46 (AUC = 0.830) and hsa_circ_0079480 (AUC = 0.9342) in PB are valuable biomarkers for AML diagnosis [77, 175]. In addition, circRNAs have been verified to be enriched in serum exosomes and have implications for early tumour diagnosis [178]. Circ_0004136 and hsa_circ_0009910 were reported to be highly expressed in exosomes secreted by AML cells, but regrettably, their diagnostic value was not specifically assessed by the study authors [141, 148]. Exosome detection has emerged as a promising method for liquid biopsy in tumour diagnosis with the advantage of minimal invasiveness [179]. However, research on exosomal circRNAs in AML is relatively scarce. In brief, we hope that circRNAs can serve as effective biomarkers for AML diagnosis in the future.

**Table 7:** Summary of recent studies on circRNAs as diagnostic markers in AML

CircRNAs	Cohort size	Dysregulation	Sample source	AUC	Sensitivity	Specificity	References
Hsa_circ_0004277	Normal (n = 8); ND AML (n = 67)	↓ in AML	BM	0.957	NR	NR	[70]
Circ-ANXA2 (Hsa_circ_0035559)	Normal (n = 50); AML (n = 130)	↑ in AML	BM	0.832	NR	NR	[74]
CircZBTB46 (Hsa_circ_103104)	Normal (n = 9); AML (n = 18)	↑ in AML	BM	0.969	NR	NR	[77]
	Normal (n = 25); AML (n = 25)	↑ in AML	PB	0.83	NR	NR	[77]
Hsa_circ_0044907	Normal (n = 45); AML (n = 45)	↑ in AML	BM	0.9447	77.78%	88.89%	[129]
CircPLXNB2 (Hsa_circ_0001257)	Normal (n = 15); AML (n = 40)	↑ in AML	BM	0.8525	NR	NR	[138]
Hsa_circ_0001947	Normal (n = 15); ND AML (n = 59)	↓ in AML	BM	0.8911	93.33%	73.33%	[152]
Circ-ANAPC7	IDA Controls (n = 80); ND AML (n = 144)	↑ in AML	BM	0.915	NR	NR	[173]
Circ-PVT1	Normal (n = 30); AML (n = 68)	↑ in AML	BM	0.92	72.10%	96.70%	[174]
	Non-proliferative haematological disorder controls (n = 30); AML (n = 68)	↑ in AML	BM	0.814	72.10%	90.00%	[174]
	Non-CR (n = 19); CR (n = 49)	↑ in non-CR	BM	0.712	57.10%	84.20%	[174]
Hsa_circ_0079480	Normal (n = 160); AML (n = 236)	↑ in AML Serum	PB	0.9342	NR	NR	[175]
Circ-Foxo3	Normal (n = 24); AML (n = 116)	↓ in AML	BM	0.633	62.10%	75%	[176]
Circ_0059706	Normal (n = 33); AML (n = 100)	↓ in AML	BM	0.925	NR	NR	[177]

↑ and ↓ indicate upregulation and downregulation respectively. *circRNA* circular RNA, *AML* acute myeloid leukaemia, *AUC* area under the curve, *ND AML* newly diagnosed acute myeloid leukemia, *BM* bone marrow, *PB* peripheral blood, *IDA* iron-deficiency anemia, *CR* complete remission, *NR* not report

### CircRNAs as prognostic biomarkers in AML

Clinically, high recurrence rates and poor prognoses remain challenges in AML patients, especially high-risk AML patients. How to accurately stratify patients by risk profile and predict the probability of relapse at the initial visit has been a matter of intense discussion for decades. Here, we summarize several circRNAs that have significant prognostic value in AML (Table 8). For example, circ-ANXA2 is highly expressed in AML, and patients with higher circ-ANXA2 levels exhibit shorter overall survival (OS) and event-free survival (EFS), poorer risk profiles, and a lower probability of complete remission (CR) [74]. Circ_0012152 expression is significantly increased in AML patients compared with individuals without AML, and high expression of circ_0012152 is strongly associated with poor prognosis [134]. Interestingly, circ_0012152 levels were decreased in patients who achieved CR but increased again in patients who experienced relapse, indicating the great potential of circ_0012152 as a biomarker for dynamically monitoring relapse. Additionally, higher circ-PVT1 expression predicts poor outcome in AML patients, specifically, shorter OS, EFS and relapse-free survival (RFS) [146, 174]. Similarly, other circRNAs, such as hsa_circ_0001990, circTASP1, and circ-PTK2, have prognostic value in AML [73, 75, 121, 129, 138, 144, 150, 154, 172, 175, 177]. Detailed relevant information about these circRNAs is listed in Table 8. In general, circRNAs are helpful markers for evaluating the prognoses of AML patients.

**Table 8:** Summary of significant associations between circRNA and AML survival

CircRNAs	Roles	Level	AML patients	Sample source	Analytical methods	Survival	References
Hsa_circ_0001990	Oncogenic	↑ in AML	70	BM	Kaplan–Meier	OS, P = 0.021	[73]
Circ-ANXA2 (Hsa_circ_0035559)	Oncogenic	↑ in AML	130	BM	Kaplan–Meier	OS, P = 0.001; EFS, P = 0.013	[74]
CircTASP1 (Hsa_circ_406083; Hsa_circ_0007340)	Oncogenic	↑ in AML	60	PB	Kaplan–Meier	OS, P = 0.0002	[75]
Circ-PTK2 (Hsa_circ_104700; Hsa_circ_0005273)	Oncogenic	↑ in AML	40	BM	Kaplan–Meier	OS, P = 0.0001	[121]
Hsa_circ_0044907	Oncogenic	↑ in AML	45	BM	Kaplan–Meier	OS, P < 0.05	[129]
Circ_0012152	Oncogenic	↑ in AML	60	BM	Kaplan–Meier	OS, P = 0.003	[134]
CircPLXNB2 (Hsa_circ_0001257)	Oncogenic	↑ in AML	40	BM	Kaplan–Meier	OS, P = 0.0364; LFS, 0.0393	[138]
Hsa_circ_0003602 (CircSMARCC1)	Oncogenic	↑ in AML	50	BM	Kaplan–Meier	OS, P = 0.0315	[144]
Circ-PVT1	Oncogenic	↑ in AML	23	BM	Kaplan–Meier	OS, P = 0027; RFS, P = 0.047	[146]
			68	BM	Kaplan–Meier	OS, P = 0.026; EFS, P = 0.017	[174]
			68	BM	Multivariate Cox’s regression analysis	OS, P = 0.029; EFS, P = 0.043	[174]
Circ_001264	Oncogenic	↑ in AML; ↑ in AML cell-derived exosomes	50	PB	Kaplan–Meier	OS, P < 0.01	[150]
Circ_0040823	Antitumour	↓ in AML; ↓ in AML serum	68	PB	Kaplan–Meier	OS, P = 0.029; DFS, P = 0.020	[154]
CircKLHL8	Antitumour	NR	111	BM, PB	Kaplan–Meier	OS, P < 0.001; DFS, P < 0.001; EFS, P < 0.001	[172]
CircSMC1 A	Antitumour	NR	111	BM, PB	Kaplan–Meier	OS, P = 0.004; DFS, P = 0.002; EFS, P = 0.02	[172]
CircFCHO2	Antitumour	NR	111	BM, PB	Kaplan–Meier	OS, P = 0.003; DFS, P = 0.02; EFS, P = 0.01	[172]
CircCFLAR	Antitumour	NR	111	BM, PB	Kaplan–Meier	OS, P = 0.003; DFS, P = 0.03; EFS, P = 0.03	[172]
Hsa_circ_0079480	Oncogenic	↑ in AML serum	236	PB	Kaplan–Meier	OS, P < 0.05; RFS, P < 0.05	[175]
			236	PB	Multivariate Cox proportional hazards regression analysis	OS, P = 0.009; RFS, P = 0.002	[175]
Circ_0059706	Antitumour	↓ in AML	57	BM	Kaplan–Meier	OS, P = 0.047	[177]

↑ and ↓ indicate upregulation and downregulation respectively. *circRNA* circular RNA, *AML* acute myeloid leukemia, *BM* bone marrow, *PB* peripheral blood, *OS* overall survival, *EFS* event-free survival, *LFS* leukaemia-free survival, *RFS* relapse-free survival, *DFS* disease-free survival, *NR* not report

### CircRNAs as therapeutic targets or agents in AML

Drug resistance and disease recurrence remain the major obstacles in AML therapy. The identification of novel therapeutic targets and optimization of treatment strategies are urgently needed to improve the clinical outcomes of AML patients. Due to their extensive regulatory roles in various cellular processes, circRNAs are hypothesized to be valuable potential therapeutic targets, and interference with circRNA expression may be a promising avenue for treating cancer.

Considering that many circRNAs are upregulated in AML, RNA-based strategies for circRNA knockdown, such as RNA interference (RNAi), antisense oligonucleotide (ASO), and CRISPR/Cas approaches, are considered particularly suitable treatment methods because they can be delivered directly to the bloodstream [180–183]. Currently, RNAi molecules can be artificially designed and synthesized in the laboratory and delivered to cells via lipid nanoparticles, exosomes, polymers and other appropriate materials [184, 185]. In AML, several mouse models with circRNA deficiency (e.g., circPLXNB2, circ_0035381, and circ_0001187) have been established using RNAi technology to verify the functions of these circRNAs [138, 149, 156]. Nonetheless, rapid degradation, low intracellular delivery efficiency, immune responses and off-target effects remain to be overcome in practice [182]. Compared with RNAi, ASOs have the advantages of better cleavage efficiency and fewer off-target effects [181]. The CRISPR/Cas system is a powerful genome-editing tool that effectively impedes circRNAs biogenesis [186]. Zheng et al. revealed that silencing of circHIPK3 through the CRISPR/Cas9 system strongly inhibited human cell growth [187]. Gu et al. reported that circIPO11 deficiency induced using CRISPR/Cas9 technology apparently suppressed the progression of chemically induced liver carcinogenesis [188]. Notably, recent studies have demonstrated that CRISPR-Cas13 systems can effectively discriminate circRNAs from their cognate mRNAs and increase their silencing efficiency, which may serve as a useful tool for the functional study of circRNAs [180, 189].

For circRNAs that are downregulated in AML, overexpression can be achieved by cloning the circRNA into lentivirus or adeno-associated virus (AAV) vectors and conjugating the vector with nanoparticles or lipid carriers to drive cell type-specific expression [190–193]. For example, using recombinant AAV9 vectors, Zeng et al. constructed a circMap3k5-overexpressing mouse model to determine the ability of circMap3k5 to alleviate intimal hyperplasia [193]. Moreover, Meganck et al. developed recombinant AAV vectors carrying transgene cassettes with intronic sequences and verified their ability to promote circRNA expression in organs such as the heart, liver and brain in mice [190]. This study highlights the possibility of precise interventions targeting circRNAs in specific tissues to improve therapeutic outcomes. However, whether linear byproducts generated during circRNA overexpression exert detrimental effects on cells requires further investigation.

CircRNAs can interact with miRNAs or proteins and subsequently participate in regulating AML pathology [22, 23]. Taking advantage of circRNAs to target suppressive/oncogenic miRNAs or proteins may contribute to AML therapy. For protein-coding circRNAs, strategies such as antibody-mediated targeting or IRES insertion upstream of ORFs may provide novel therapeutic avenues [62, 194]. Taken together, these findings imply that altering circRNA expression levels may provide new strategies for AML treatment.

Moreover, circRNA vaccines also show great promise for AML therapy. Compared with normal mRNA vaccines, circRNA vaccines produce higher concentrations of antigens for a longer time because of their greater stability [195]. Qu et al. demonstrated that a novel circRNA vaccine encoding the antigen of SARS-CoV-2 effectively promoted immune activation in mice and rhesus macaques upon infection with SARS-CoV-2 [195]. In the area of cancer research, Li et al. encapsulated the synthetic circRNA^OVA−luc^, which encodes the restricting H2-Kb peptide OVA 257–264 and luciferase, into lipid nanoparticles to construct a circRNA vaccine and verified its antitumour effect in a variety of tumour-bearing mouse models, including colorectal carcinoma, orthotopic melanoma, and lung metastasis melanoma mouse models [196]. This circRNA vaccine triggered robust innate and adaptive antitumour immune activation in multiple mouse tumour models and showed superior antitumour efficacy [196]. Huang D et al. reported that vaccination with circFAM53B efficiently elicited antitumour immunity in an antigen-specific manner by encoding cryptic peptides and significantly inhibited tumour growth in a B16 F10 mouse melanoma model [197]. Although there is a lack of relevant studies on circRNA vaccines in AML, we believe that significant advances will be made in the coming years.

### Clinical application prospects of circRNAs in AML

Although noncoding RNAs (ncRNAs) were previously considered noise in genomic transcription, their functions have become popular research topics in recent years, paving the way for their clinical application; more than 1000 miRNA-related clinical trials and more than 100 lncRNA-associated clinical trials have been registered in the ClinicalTrials.gov database (https://www.clinicaltrials.gov/). However, no circRNA-related clinical trials were found in this database. Several miRNA mimics or inhibitors have successfully entered clinical trials. For example, MRG-106, an inhibitor of miR-155, exhibits excellent antitumour efficacy without serious adverse reactions in diffuse large B-cell lymphoma (Registration ID: NCT02580552) [198]. Moreover, a miR-34a mimic (MRX34) [199], a miR-16 mimic (TargomiR) [200], and a miR-193-3p mimic (INT-IB3) have also been tested in clinical trials. For lncRNAs, clinical treatment approaches involving targeting lncRNAs are still lacking, although their possible use as tumour biomarkers has gained more attention. For example, Fayoum University recently completed a clinical trial that explored the clinical utility of the salivary expression of the lncRNA MALAT1 in the diagnosis of oral squamous cell carcinoma (Registration ID: NCT05708209). Assiut University conducted a clinical trial to evaluate the relationship between lncRNA CCAT1 and tumour staging in patients with colorectal cancer and its diagnostic value (Registration ID: NCT04269746). In addition, a clinical trial at Strasbourg University Hospital is recruiting volunteers to investigate the prognostic value of the lncRNA MFI2-AS1 in localized clear cell kidney cancers (Registration ID: NCT04946266).

Although this field of research is in its nascent stage, recent studies have demonstrated that circRNAs are characterized by high abundance, relative stability, and evolutionary conservation and are closely related to the development and progression of various diseases, making them ideal biomarkers for tumour diagnosis, prognostic assessment, and therapy [14, 16, 17, 20, 22, 23]. Nevertheless, no circRNAs are yet implemented in clinical practice, and we believe that major developments can be anticipated in the future. However, limited circRNA-specific target sites, low delivery efficiency, poor specificity and tolerability, toxicity and off-target effects are still major obstacles to the clinical application of circRNAs. Overall, inhibiting the activity of oncogenic circRNAs or overexpressing tumour-suppressor circRNAs can be beneficial treatment approaches, but some technical limitations and challenges still exist.

## Recommendations and future perspectives

Although we have gradually elucidated the specific functions of circRNAs, our current understanding may represent just the tip of the iceberg, and numerous issues still need to be addressed to move the field forwards. First, there is still no generally established consensus for circRNA nomenclature, and there is a lack of universal and comprehensive circRNA-associated public databases. These problems make it difficult for investigators to obtain exact genomic locations and detailed information from databases using circRNA names. Second, superior detection methods, such as single-cell spatial noncoding transcriptomics, nanopore-based sequencing, and electrochemical detection techniques, are needed to identify and quantify circRNAs. Third, the off-target effects of circRNA knockdown must be considered carefully due to the high sequence similarities between circRNAs and their cognate mRNAs. Recent studies have demonstrated that CRISPR–Cas13 systems can effectively discriminate circRNAs from their cognate mRNAs and increase their silencing efficiency, making these systems useful tools for the functional study of circRNAs [180, 189]. Moreover, the inefficiency of circularization when circRNAs are overexpressed is an inevitable problem. Fourth, most circRNA research in AML has focused on the role of circRNAs as miRNA sponges, but the specific underlying mechanisms remain to be further elucidated, and other mechanisms also deserve investigation. Fifth, the functions and downstream mechanisms of circRNAs have attracted much attention, but the modes of circRNA biogenesis, spatial structure, transportation, degradation, and chemical modifications, in addition to m^6^A modifications, have been much less well studied. Sixth, circRNAs related to stem cell phenotype or function have received less attention. Seventh, the clinical translation of circRNA-based therapies still faces challenges. As a whole, research on circRNAs remains in its infancy, and associated limitations and challenges need to be addressed.

## Conclusions

AML is a challenging and biologically complex disease that is driven, in part, by genetic mutations, heterogeneous clones and epigenetic alterations, and our current knowledge of its pathogenesis is insufficient [201, 202]. Previously, circRNAs were considered functionless byproducts of RNA mis-splicing [203, 204]. However, circRNAs are now considered emerging molecular regulators of various physiological and pathological processes. Many circRNAs with great physiological and clinical significance have been identified to be specifically expressed in AML, making them attractive candidate diagnostic, prognostic and therapeutic targets [22, 23]. Herein, we have summarized the biogenesis, categories, degradation, regulatory mechanisms and functions of circRNAs, with an emphasis on elucidating dysregulated circRNAs in AML and their clinical implications. Moreover, we outlined a series of online databases and tools for circRNA exploration, which can provide important guidance for subsequent studies. Although several key challenges remain, we believe that circRNAs may be developed as clinical diagnostic and prognostic markers, therapeutic targets, and even RNA drugs in the future.

## Data Availability

No datasets were generated or analysed during the current study.

## Abbreviations

AML: Acute myeloid leukaemia
circRNA: Circular RNA
Bcl-2: B-cell lymphoma-2
FLT3: FMS-like tyrosine kinase-3
IDH: Isocitrate dehydrogenase
CAR-T: Chimeric antigen receptor-modified T
pre-mRNAs: Precursor messenger RNAs
ecircRNAs: Exonic circRNAs
EIciRNAs: Exon‒intron circRNAs
ciRNAs: Intronic circRNAs
RBP: RNA binding protein
f-circRNAs: Fusion circRNAs
tricRNAs: TRNA intronic circular RNAs
mecciRNAs: Mitochondria-encoded circRNAs
rt-circRNAs: Read-through circRNAs
pretRNAs: Precursor tRNAs
Ago2: Argonaute2
miRNAs: MicroRNAs
lncRNAs: Long non-coding RNAs
MREs: MiRNA response elements
MLL-r: Mixed lineage leukaemia-rearranged
IRESs: Internal ribosome entry sites
m6 A: N6-methyladenosine
UTR: Untranslated region
ORF: Open reading frame
chr: Chromosome
EMI: Extramedullary infiltration
NPM1: Nucleophosmin
HSPC: Healthy haematopoietic stem and progenitor cell
CCLE: Cancer cell line encyclopedia
FAB: French-American-British
BM: Bone marrow
PB: Peripheral blood
WBC: White blood cell
PLT: Platelet
HGB: Haemoglobin
AUC: Area under the curve
OS: Overall survival
EFS: Event-free survival
LFS: Leukaemia-free survival
RFS: Relapse-free survival
DFS: Disease-free survival
